# Mortality due to Japanese oak wilt disease and surrounding forest compositions

**DOI:** 10.1016/j.dib.2015.08.017

**Published:** 2015-08-29

**Authors:** Michio Oguro, Sawako Imahiro, Shoichi Saito, Tohru Nakashizuka

**Affiliations:** a Graduate School of Life Sciences, Tohoku University, Aoba 6-3, Sendai, Miyagi 980-8578, Japan; b Yamagata Prefectural Forest Research and Instruction Center, 2707 Sagae-Hei, Sagae, Yamagata 991-0041, Japan

**Keywords:** Japanese oak wilt, *Raffaelea quercivora*, *Platypus quercivorus*, *Quercus crispula*, *Quercus serrata*, Landscape

## Abstract

Japanese oak wilt (*Raffaelea quercivora*) is a vector-borne disease transmitted by the flying ambrosia beetle, *Platypus quercivorus*, and causes mass mortality in the fagaceous species of Japan. The data described in this article are available in Mendeley Data, DOI: 10.17632/xwj98nb39r.1 [1] and include the mortality status of 1089 *Quercus crispula* and 846 *Quercus serrata* trees and surrounding forest conditions. The findings using this dataset were published in M. Oguro, S. Imahiro, S. Saito, T. Nakashizuka, Relative importance of multiple scale factors to oak tree mortality due to Japanese oak wilt disease, For. Ecol. Manag. (2015) doi:10.1016/j.foreco.2015.07.016 [2].

## Specifications table

**Table:** 

Subject area	Ecology
More specific subject area	Forest pathology
Type of data	Text file
How data was acquired	Field observation and geoprocessing of existing data.
Data format	Raw
Experimental factors	N/A
Experimental features	N/A
Data source location	Tsuruoka, Yamagata, Japan. Locations of field observation are included in the dataset.
Data accessibility	Data are provided in Mendeley Data, DOI: 10.17632/xwj98nb39r.1

## Value of the data

• This data provides field-observed viability status of 1935 trees and surrounding stand conditions.
• This data also includes landscape factors with two spatial scales.
• This data is useful for analyzing relationships between forest conditions and disease occurrence. Such information is crucial for effective disease management.

## 1 Data

The data described in this article are available in Mendeley Data, DOI: 10.17632/xwj98nb39r.1

These data are: (1) viability status (dead/alive), (2) basal area, (3) species compositions of stands around the tree, and (4) landscape metrics around the tree for individuals of *Quercus crispula* and *Quercus serrata*. These data are shown in “*data.csv*”. For detailed explanation of each column in the files, please see Table 1.

## 2 Experimental design, materials and methods

### 2.1 Field observations at the stand level

**Fig. 1:**
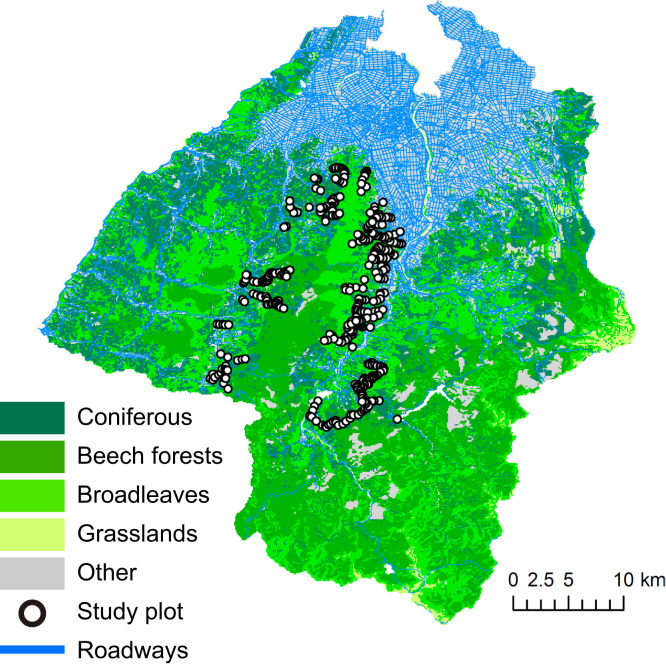
Vegetation map of the study region (Tsuruoka city). Location of the study plots is provided in “gps_points.csv” in [1].

From June to early July of 2009, we traveled all forest roads that could be accessed by car in the former Asahi Village, Kushibiki Town, and Nurumi Town areas of Tsuruoka City (Fig. 1). These forest roads were selected to cover the variation in land-use types and vegetation, and because of their accessibility. Land-use types found in the study region include secondary forest dominated by *Q. crispula*, beech forest, coniferous plantations of *Cryptomeria japonica*, *Pinus densiflora,* and *Larix kaempferi*, rice fields, and residential areas.

Every 200 m along the forest roads, we searched for the nearest *Q. crispula* or *Q. serrata* individual and recorded its location using a Global Positioning System (GPS) device. Locations of each study plot were noted in “*gps_points.csv*”. We established a circular plot with a 10 m radius around each tree, and identified all trees with a diameter at breast height (DBH) of >10 cm within that area. *Q. crispula* stems with a diameter of <10 cm are rarely attacked by *Platypus quercivorus* [3]. The DBH of each tree in each plot was measured, and trees were grouped into three classes according to size: 10–30 cm, 30–50 cm, and >50 cm. For all *Q. crispula* and *Q. serrata* individuals in the plot, we also recorded viability status (dead or alive). Overall, a total of 365 plots were surveyed, containing a total of 4482 trees, including 1089 *Q. crispula* and 846 *Q. serrata* trees. This raw observation data was included in “*trees.csv*”.

Because previous studies [4,5] showed that the size of an individual tree is positively related to the occurrence of dieback, the basal areas (BA) of individual trees were calculated using their DBH measurements. The stand-level density of host trees is known to influence the occurrence of Japanese oak wilt [5,6]. In addition, the existence of non-host trees could potentially affect disease occurrence [7,8]. Therefore, the BAs and number of individuals were summed for each of three host species (*Q. crispula*, *Q. serrata*, and *F. crenata*), and each of the three groups of non-host species (*C. japonica*, other broad-leaved and coniferous species).

### 2.2 Landscape metrics

Because it is hard to obtain detailed species composition data in a large geographic range, the species compositions of the communities in the landscape around the study plots were represented by metrics calculated from vegetation maps. All geoprocessing was conducted using ArcGIS version 10.1 (ESRI, Redlands, CA, USA). Two vegetation maps, namely, a vegetation map created using the 5th Vegetation Survey of the Basic Survey of Natural Environment Conservation by the Ministry of Environment (MOE) of Japan (http://www.biodic.go.jp/trialSystem/shpddl.html) and a map based on the Forest Planning Data provided by the Division of Forestry, in the Department of Agriculture, of the Forestry and Fisheries of Yamagata Prefecture were integrated in order to obtain land-use or land-cover data. The vegetation categories of the original maps were reclassified into five categories: (1) broad-leaved forest (dominated by the susceptible hosts, *Q. crispula* and *Q. serrata*), (2) coniferous forest (including plantations, dominated by non-host species), (3) beech forest (dominated by a resistant host, *F. crenata*), (4) grassland, and (5) other. The correspondence between original and new vegetation categories is shown in “*vegetation_maps.csv*”. As the Forest Planning Data contained more detailed data on composition and forest age, the map based on this information was overlaid on the MOE vegetation map to create a vegetation map of the study region (Fig. 1).

The coverage proportions of the five vegetation categories found in the study plots were calculated as follows: first, circular buffer zones, 100 m (small) and 1000 m (large landscape level) in radius, were created for each study plot. Then, each vegetation map was clipped at the edges of the buffer, and the proportions of the different vegetation categories within the buffer zone were calculated at both scales. To represent the existence of non-host species at the landscape level, the Shannon׳s diversity index,

$$H=-\sum_{i=1}^{s}p_{i}\;\ln\;P_{i}$$

of each study plot was also calculated at both scales, with *S* being the number of vegetation categories and *p*_*i*_ being the proportion of the area of *i*th vegetation category to the total area in the buffer zone.

The distance between each study plot and its nearest *Q. crispula* forest was calculated. For this purpose, we extracted the *Q. crispula* forest type from the MOE vegetation map and calculated each plot׳s distance from the nearest *Q. crispula* forest using the *Near* tool in ArcGIS. In addition, the altitude of each study plot was obtained from a 10 m digital elevation model (DEM) provided by the Geospatial Information Authority of Japan (http://fgd.gsi.go.jp/download/).

## Figures and Tables

**Table 1:** Explanation of each column in the data files.

File name	Column name	Unit	Description
trees.csv	gps_point	–	ID of the study plot.
	dbh	cm	Diameter breast height of the tree.
	type	–	Type of the tree.
	dead	–	Viability status of the tree. TRUE: dead, FALSE, alive.
	species	–	Species name.

data.csv	point	–	ID of the study plot.
	species	–	crispula: *Q. crispula*, serrata: *Q. serrata*.
	dead	–	Viability status of the tree. TRUE: dead, FALSE: alive.
	ba	cm^2^	Basal area of the tree.
	n.serrata	–	Number of *Q. serrata* in the plot of 10 m radius around the tree.
	n.cryptomeria	–	Number of *C. japonica* in the plot of 10 m radius around the tree.
	n.fagus	–	Number of *F. crenata* in the plot of 10 m radius around the tree.
	n.crispula	–	Number of *Q. crispula* in the plot of 10 m radius around the tree.
	n.broad	–	Number of broadleaved trees excluding *Q. serrata*, *Q. crispula*, and *F. crenata* in the plot of 10 m radius around the tree.
	n.conifer	–	Number of coniferous trees excluding *C. japonica* in the plot of 10 m radius around the tree.
	ba.serrata	cm^2^	Total basal area of *Q. serrata* in the plot of 10 m radius around the tree.
	ba.cryptomeria	cm^2^	Total basal area of *C. japonica* in the plot of 10 m radius around the tree.
	ba.fagus	cm^2^	Total basal area of *F. crenata* in the plot of 10 m radius around the tree.
	ba.crispula	cm^2^	Total basal area of *Q. crispula* in the plot of 10 m radius around the tree.
	ba.broad	cm^2^	Total basal area of broadleaved trees excluding *Q. serrata*, *Q. crispula*, and *F. crenata* in the plot of 10 m radius around the tree.
	ba.conifer	cm^2^	Total basal area of coniferous trees excluding *C. japonica* in the plot of 10 m radius around the tree.
	fagus.100	–	Proportion of beech forests in the circle of 100 m radius around the tree.
	broad.100	–	Proportion of broad-leaved forests in the circle of 100 m radius around the tree.
	conifer.100	–	Proportion of coniferous forests including plantations of *C. japonica* in the circle of 100 m radius around the tree.
	grass.100	–	Proportion of grasslands in the circle of 100 m radius around the tree.
	shannon.100	–	Shannon׳s H of land use in the circle of 100 m radius around the tree.
	fagus.1000	–	Proportion of beech forests in the circle of 1000 m radius around the tree.
	broad.1000	–	Proportion of broad-leaved forests in the circle of 1000 m radius around the tree.
	conifer.1000	–	Proportion of coniferous forests including plantations of *C. japonica* in the circle of 1000 m radius around the tree.
	grass.1000	–	Proportion of grasslands in the circle of 1000 m radius around the tree.
	shannon.1000	–	Shannon׳s H of land use in the circle of 1000 m radius around the tree.
	altitude	m	Altitude of the plot.
	distance	m	Distance from the nearest *Q. crispula* forest (m).

gps_points.csv	point_name	–	ID of the study plot.
	longitude	°	Longitude of the study plot.
	latitude	°	Latitude of the study plot.

vegetation_map.csv	Type of the map	–	Source vegetation map used for making vegetation map of the study region.
	Original category	–	Original vegetation category used in the source maps.
	New category	–	New vegetation category used in the vegetation map of the study region.
	Japanese name	–	Original vegetation category used in the source maps in Japanese.
